# Probing Metagenomics by Rapid Cluster Analysis of Very Large Datasets

**DOI:** 10.1371/journal.pone.0003375

**Published:** 2008-10-10

**Authors:** Weizhong Li, John C. Wooley, Adam Godzik

**Affiliations:** 1 California Institute for Telecommunications and Information Technology, University of California San Diego, La Jolla, California, United States of America; 2 Center for Research in Biological Systems, University of California San Diego, La Jolla, California, United States of America; 3 Burnham Institute for Medical Research, La Jolla, California, United States of America; University College London, United Kingdom

## Abstract

**Background:**

The scale and diversity of metagenomic sequencing projects challenge both our technical and conceptual approaches in gene and genome annotations. The recent *Sorcerer II* Global Ocean Sampling (GOS) expedition yielded millions of predicted protein sequences, which significantly altered the landscape of known protein space by more than doubling its size and adding thousands of new families (Yooseph et al., 2007 PLoS Biol 5, e16). Such datasets, not only by their sheer size, but also by many other features, defy conventional analysis and annotation methods.

**Methodology/Principal Findings:**

In this study, we describe an approach for rapid analysis of the sequence diversity and the internal structure of such very large datasets by advanced clustering strategies using the newly modified CD-HIT algorithm. We performed a hierarchical clustering analysis on the 17.4 million Open Reading Frames (ORFs) identified from the GOS study and found over 33 thousand large predicted protein clusters comprising nearly 6 million sequences. Twenty percent of these clusters did not match known protein families by sequence similarity search and might represent novel protein families. Distributions of the large clusters were illustrated on organism composition, functional class, and sample locations.

**Conclusion/Significance:**

Our clustering took about two orders of magnitude less computational effort than the similar protein family analysis of original GOS study. This approach will help to analyze other large metagenomic datasets in the future. A Web server with our clustering results and annotations of predicted protein clusters is available online at http://tools.camera.calit2.net/gos under the CAMERA project.

## Introduction

The vast majority of microbes cannot be grown in pure cultures. However, advances in sequencing technology now allow us to study such microbes directly in their environment without isolation and culturing. The new science of metagenomics studies the microbes under many different environmental conditions such as soil, animal guts, marine and other water body [1]–[8]. The largest metagenomic study to date is the *Sorcerer II* Global Ocean Sampling (GOS) expedition [1], [2]. The first leg of this trip sampled 41 locations from the northwestern Atlantic through the eastern tropical Pacific and obtained nearly 8 million environmental DNA reads. Such studies, with the great scale and diversity of data, challenge both our technical and conceptual approaches in gene and genome annotations.

The raw sequence reads from a typical metagenomic study usually cannot be assembled into full genomes since a single sampling does not produce enough coverage required by assembly programs. This failure is especially severe for the organisms at low population densities. Another assembling problem is posed by the high sequence similarities among closely related species. The available whole-genome-tested gene prediction programs work poorly on short and fragmented sequences, the use of such programs results in a significant underprediction of proteins. And a significant overprediction arises from predicting proteins by translating all 6 reading frames, a method used in the GOS study in Open Reading Frames (ORFs) calling. So, among the 17.4 milliion ORFs identified in the GOS study, many are spurious ORFs, which are not protein coding sequences. In addition, in order to accommodate the partial DNA sequences, a GOS ORF starts at either a start codon or the start of the DNA sequence, and ends at either a stop codon or the end of the DNA sequence. So the GOS ORFs are also fragmented.

Recently, several studies, such as simulated datasets[9], comparative sequence analysis [10], taxonomy [11], [12], statistical comparison [13], functional diversity analysis [14], and binning [15], were reported to address the metagenomics-specific problems. Despite these problems, metagenomics offers a fresh view of the protein universe and classification of protein families. A single GOS study more than doubled the number of known protein sequences. Even more sequences will flow from ongoing and future metagenomic projects. Clustering analysis, which groups similar sequences into clusters or families, provides a first glimpse into the internal structure of the metagenomic datasets and identifies novel families. In metagenomics, clustering plays another important role as a tool to help to recognize some of the spurious ORFs that are introduced in the initial ORF calling stage, thus providing an error-correction mechanism.

Various clustering approaches have been applied to protein family analysis [16]–[24]. However, most existing applications start from an all-by-all sequence comparison, which is very computationally intensive and almost impossible to do for very large sets. Thus, most existing approaches can handle only rather small datasets or have to rely on incremental buildup of families. In order to cluster the GOS dataset using such approaches, extraordinary resources are needed. In the clustering study by Shibu and co-workers [2], the all-by-all comparison took over 1 million CPU hours (>1 year on a 100-CPU cluster). Given the computational requirements, it is very difficult to test the robustness of the clustering method, for instance, by using a different set of BLAST parameters in calculating sequence similarities.

In the past few years, we have developed an ultra-fast protein sequence clustering algorithm, CD-HIT [25]–[27], which has been applied widely in sequence analyses such as preparing the UniRef [28] database in UniProt [29]. Several new features introduced in the latest version of CD-HIT made it especially useful for its application for very large metagenomic datasets. In this study, we used a CD-HIT-based clustering approach to analyze GOS ORFs for several specific goals:

- To study the internal structure of the ocean metagenomes, such as the distribution of protein families and their functions and the distributions of families among different samples.
- To identify novel protein families.
- To reduce efforts in sequence annotation by annotating clusters instead of each individual ORF.
- To help to recognize spurious ORFs by analyzing the internal structure of clusters.

## Results

### Sequence clustering

**Figure 1:**
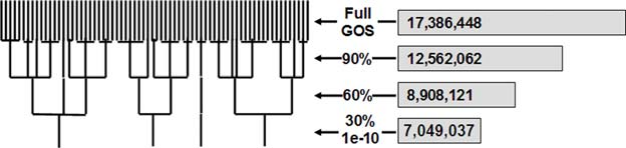
Step-wise clustering of GOS ORFs.

The GOS study identified 17,386,448 ORFs of at least 60 amino acids [1], [2]. Based on the average coding density in Bacteria and Archaea, we can estimate that at least half of these ORFs are spurious. One of our goals is to identify such ORFs by specific features of their clusters. The clustering was performed by the program CD-HIT [25]–[27] using a newly added accurate mode based on full-length or nearly full-length sequence similarities. Since the majority of GOS ORFs are partial sequences, we allowed a short sequence to be clustered with a long sequence if it was completely contained within the latter. Three clustering steps at different similarity thresholds (90%, 60%, and 30% identity) were performed one after another. In each subsequent step, only the representative sequences of clusters generated in the previous step were used (Figure 1). This hierarchical clustering approach not only automatically produced a treelike structure, but also maximized the computational efficiency and quality of clustering. Detailed settings of the clustering procedure are described in the Methods section.

In the first step, the clustering threshold, which is the similarity cut-off within a cluster, was set at a sequence identity ≥90% in order to eliminate redundant (nearly identical) sequences. The identity threshold in the second step was set at ≥60%, and the threshold in the third step was set to include any sequence meeting either a sequence identity ≥30% or an expect value ≤1e-10. We used conservative clustering thresholds to ensure that each cluster contained relatively closely related sequences. With more sensitive tools such as PSI-BLAST [30], PDB-BLAST [31], Hidden Markov Models (HMMs) [32], and FFAS [33], many clusters could further be identified as related and be grouped together. Therefore, we expect that the actual number of families in the GOS set is much smaller. The reason why we did not proceed with further clustering is that the presence of many spurious ORFs, which have an uncommon amino acid composition or pattern, led to unusual behavior in the profile-based methods.

The first-step clustering identified 12,562,062 non-redundant sequences from the original 17,386,448 GOS ORFs and yielded a 28% reduction rate (Figure 1). When we mention the “size of a cluster” in the rest of this paper, it refers to the number of the non-redundant sequences in that cluster. The second and third steps resulted in 8,908,121 and 7,049,037 clusters with 49% and 60% reduction rates. As a reference, we performed the same clustering procedure on the NCBI NR dataset of February 2006, which contained 3,289,889 sequences. The numbers of clusters at the 90%, 60%, and 30% levels were 2,104,938, 1,333,002, and 462,965, which corresponded to the 37%, 66%, and 86% database reduction.

**Figure 2:**
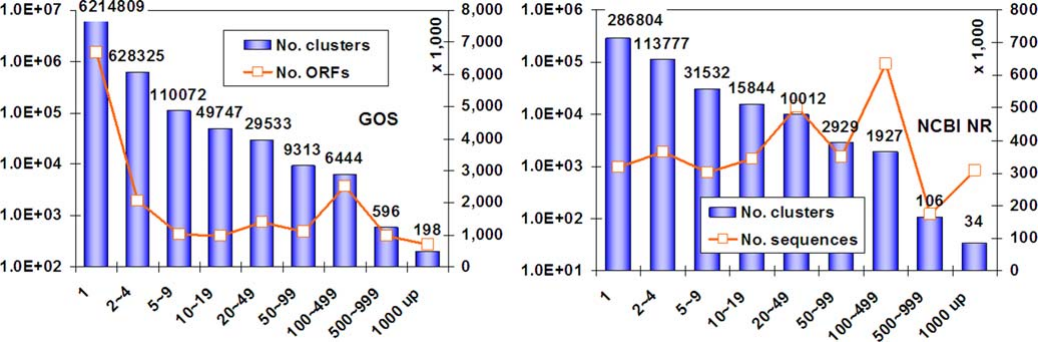
Distribution of clusters of GOS ORFs and NCBI NR proteins. The *x*-axis is the size of a cluster defined by the number of non-redundant sequences at 90% identity. Blue bars with numbers plotted against the left *y*-axis in log scale show the numbers of clusters. Red line plotted against right *y*-axis show the number of corresponding ORFs or sequences. Left is for GOS, and right is for NCBI NR.

The distribution of clusters identified in the last step is plotted in Figure 2. There are noticeable differences between the internal structures of GOS and NR, the most obvious difference being the unusually large amount of singletons within the GOS ORFs. Almost certainly, the majority of these singleton ORFs are spurious ORFs. Such ORFs were not clustered because of their random characters. If we exclude singletons and very small clusters of sizes 2–4, then the overall clustering behavior of GOS ORFs and NR are very similar in terms of fraction of clusters binned by size, sequence distribution pattern, and power law distribution curve, despite the significant organism composition difference between GOS and NR and the fact that GOS ORFs are mostly fragments. For example, the largest size bin corresponds to sizes 100–499 in both databases.

Clustering described in this study was performed on a 16-processor Linux cluster. The CPU times for the three steps are 135, 1,224 and 8,200 hours, respectively. The total CPU cost was about two orders of magnitude less than the clustering effort in the original GOS study [2].

### Protein enrichment

One of the goals of our clustering analysis was to enrich the real genes into clusters. One way of testing the enrichment is to check the ORFs with strong evidence of being real proteins. A total of 3.70 million GOS ORFs match HMM models from either Pfam or Tigrfam [2]. These HMM-validated ORFs were used as benchmarks for gene-enrichment estimation.

A total of 3.30 million (89%) HMM-validated ORFs were found in 46,084 clusters of size ≥20. Thus, clustering placed most genuine ORFs in large clusters as expected. About 0.14 million (4%) such ORFs were found in 49,747 clusters of sizes 10–19. An additional 4% of HMM-validated ORFs are found in clusters of sizes 2–9. Nearly 3% of these ORFs are in singletons, most of which have significant but not full-length similarity to other ORFs, so they could not be clustered. These ORFs may have novel domain structure, but more likely, they are just partially correct sequences with errors such as frame shift and incorrect boundaries and should be excluded in further analysis.

### Predicted protein clusters

We focus on the 46,084 large clusters of size ≥20, which contain 6,652,534 (38%) ORFs. These clusters include ∼90% of all the protein-coding ORFs (estimated according to the result of protein enrichment). Analyzing large clusters allows us to take advantage of combined information of all homologous sequences within that cluster to reach more confident results. To proceed, first we need to detect and filter out the spurious ORF clusters. In the original GOS study, ORFs that overlap with other more protein-like ORFs on different reading frames were detected and called shadow ORFs [2]. Here, we introduce an independent method to detect spurious ORFs based on sequence consensus of homologous proteins (details are described in the Methods section). Most known protein families, even very large and diverse ones, display conservation patterns along the sequences, which usually form blocks within multiple alignments. Such patterns are the result of evolutionary pressure to maintain the structures and function of proteins. The conserved residues are related with hydrophobic cores, conserved catalytic residues, and functional motifs. Clusters that lack such patterns are very likely translations from non-protein-coding frames.

We define an HMM-validated cluster as having at least one-half of the ORFs match the same Pfam or Tigrfam family. In total, 13,333 large clusters are HMM-validated. Our sequence consensus-based method identified 33,043 protein-like clusters, which include almost all (13,006, or 97.5%) the HMM-validated clusters. Only 327 HMM-validated clusters were missed. Overall, we preserved nearly all the protein-coding clusters while removing a big fraction of spurious protein clusters. We combined the 33,043 protein-like clusters and 327 HMM-validated clusters that were missed for a total of 33,370 and used them for further analysis. These clusters are called predicted protein clusters, which contain 5,992,629 (34%) ORFs.

**Figure 3:**
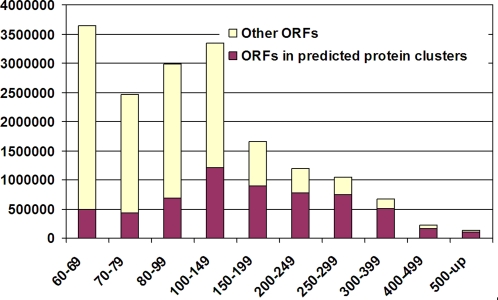
Distribution of ORFs by length. The *x*-axis is the length bin of ORFs. The *y*-axis is number of ORFs in two groups: ORFs in predicted protein clusters and other ORFs.

Figure 3 shows the distribution of ORFs by length. As expected, since short ORFs contain more spurious predictions, we observed that as the length increases more ORFs fall in predicted protein clusters.

### Novelty of predicted protein clusters

For the 33,370 predicted protein clusters, we first ran BLASTP [30] to search for their homologs from NCBI NR of January 2007 using the representative sequences selected by the CD-HIT program as queries. For the clusters without any BLASTP hits, we applied slower but more sensitive homology detection tools PDB-BLAST[31] and FFAS[33] to search for more remote homologs. With PDB-BLAST, we searched the same NR database, and with FFAS, we searched three databases, PDB, Pfam, and COG, provided by the FFAS developers.

**Figure 4:**
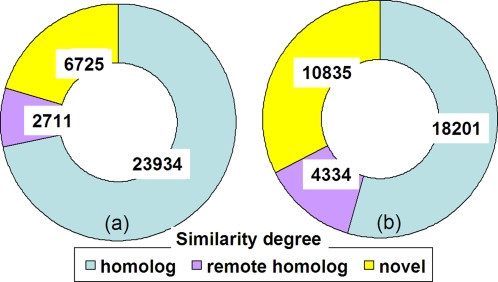
Pie chart of the predicted GOS protein clusters. The predicted GOS protein clusters are in three classes by similarities to existing protein sequences in NR: with homolog (BLASTP hits), with remote homolog (PDB-BLAST or FFAS hits), and novel (no hit). All matches to proteins in NR are considered in (a). Only matches to at least 20 non-redundant sequences in NR are included in (b).

We classified clusters into three types: having homologs (BLASTP matches), having remote homologs (PDB-BLAST or FFAS matches), or having no recognizable homologs in the known protein space. A match needs to cover at least 60 residues or 50% of the length of the query with an expect value ≤1e-3 for BLASTP and PDB-BLAST, or score ≤−9.5 for FFAS. We have 23,934, 2,711, and 6,725 clusters in these three categories, respectively (Figure 4a). Actually, many of the “known” clusters (with homologs or remote homologs) only match a few hypothetical proteins in NR. So they are really unknown in terms of function. If we require a cluster to have at least 20 homologs or to be a “known” cluster, then the GOS dataset has 10,835 novel clusters (Figure 4b).

These novel clusters are very interesting and they are valuable materials for discovery of new functions. However, they may still possibly contain spurious ORFs despite having protein-like conservation patterns. As homology information cannot be used to predict their function, additional information such as genomic context may help (see the next section), but such analysis is difficult to do in a high-throughput mode. However, with today's technology, some of them can even be experimentally validated. Five crystal structures from these novel families were solved by our collaborators from the Joint Center for Structural Genomics and deposited in PDB (PDB IDs: 2OD5, 2OD4, 2OD6, 2OP5, 2PGC).

### Genomic context analysis

One problem in the analysis of metagenomic data is the fragmented nature of the sequences. For example, the average length of GOS assemblies is only ∼1,500 bp. Despite this, information such as relative gene positions on genomic contigs can still be very valuable in gene annotation. Here, we define a simple neighboring relation between any ORF that ends with a stop codon and its next non-overlapping ORF within 500 bp on the same strand. We found 7,244,944 such pairs of ORFs, and in addition, we identified 10,436 pairs of clusters where at least 20 or one third of the ORFs in one cluster are genomic neighbors of the ORFs in another cluster. Since one cluster can be a neighbor to multiple clusters, which may correspond to alternative gene organization in different species, we observed some more complicated neighboring relations.

For example, we found 17,270 cases where 3 clusters could form an operon-like structure, and 31,726 cases where 4 clusters could form an operon-like structure. Detailed analyses of such relations will be the subject of a separate study. For the 6,725 novel clusters, 2,089 can be assigned to such “virtual operons,” including 1,247 that are next to characterized clusters, providing possible hints to their functions.

### Organism composition and functional class

**Figure 5:**
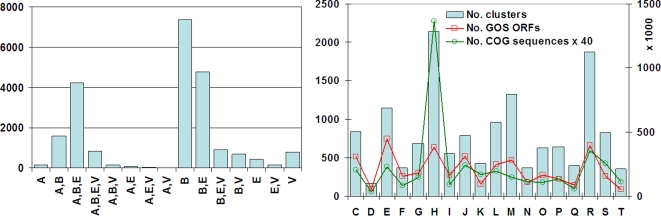
Distribution of clusters by their associated organisms and functional classes. The left figure shows the number of clusters by organisms at the level of main domains of life (Archea, Eucaryota, Bacteria, and Viral). For example, “A,B” means a cluster has only Archaea and Bacteria homologs. The right figure shows distributions by COG functional classes. Blue bars plotted against left *y*-axis show numbers of clusters. Red and green lines plotted against right *y*-axis are numbers of GOS ORFs and the underlying COG sequences multiplied by 40 for scaling. COG functional classes are: C, energy; D, cell division, chromosome partitioning; E, amino acid; F, nucleotide; G, carbohydrate; H, coenzyme; I, lipid; J, translation, ribosomal structure, and biogenesis; K, transcription; L, DNA replication, recombination, and repair; M, cell wall/membrane/envelope; N, cell motility and secretion; O, posttranslational modification, protein turnover, chaperones; P, inorganic ion; Q, secondary metabolites; R, general function prediction only; S, function unknown; and T, signal transduction.

For the 23,934 clusters that have BLASTP homologs in NR, we examined the distribution of the clusters in the main phylogenetic groups that their homologs belong to: Archaea, Bacteria, Eukaryota, and Viruses (Figure 5). The top three dominant classes are Bacteria only (B), Bacteria and Eukaryota (B,E), and Archea, Bacteria, and Eukaryota (A,B,E). There are 436 clusters that have only eukaryotic homologs—the “E” clusters in Figure 5. These clusters, perhaps, represent the Eukaryota component within GOS samples or perhaps, new, previously unknown bacterial or archeal homologs of families thought to be specific to eukaryotes.

We compared the GOS clusters against the Clusters of Orthologous Groups (COGs) [34] using BLASTP. For each cluster that had consistent COG hits, i.e., matches ≥5 non-redundant significant homologs from the same COG, the top matching COG was assigned to this cluster. A total of 14,481 out of 33,370 clusters were given COG assignment (Figure 5). When compared to the underlying sequences in COG database, GOS has many fewer proteins in class “H” (coenzyme transport and metabolism), but much more in classes “E” (amino acid transport and metabolism) and “M” (cell wall/membrane/envelope biogenesis).

### Distribution of clusters among samples

**Figure 6:**
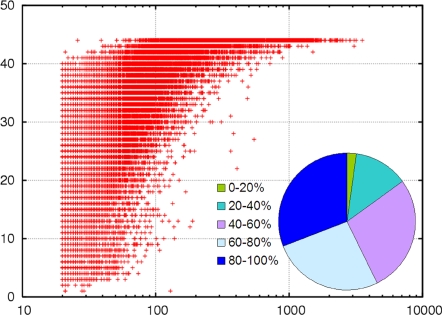
Distribution of predicted GOS protein clusters by their associated samples. The *x*-axis is the cluster size; the *y*-axis is the number of a cluster's associated samples. The pie chart inset shows distribution of clusters by the percentage of samples to which a cluster is associated.

The GOS sequences represent 44 samples taken at 41 different locations throughout the Atlantic and southern Pacific oceans. Since the GOS study used a cross-sample assembly strategy, a single assembly may associate with multiple samples. As a result, an ORF may also be mapped to multiple samples. We built the ORF to sample mapping by combining all the mapping information, including ORF to assembly, assembly to read, and read to sample, as provided by the GOS study [1], [2]. Then, we created a mapping table between 33,370 predicted protein clusters and 44 samples. We found that most of the clusters are associated with many samples (Figure 6). For example, 2,279 clusters are found in every sample, and 6,593 and 10,316 clusters are found in ≥90% and ≥80% of samples, respectively.

**Figure 7:**
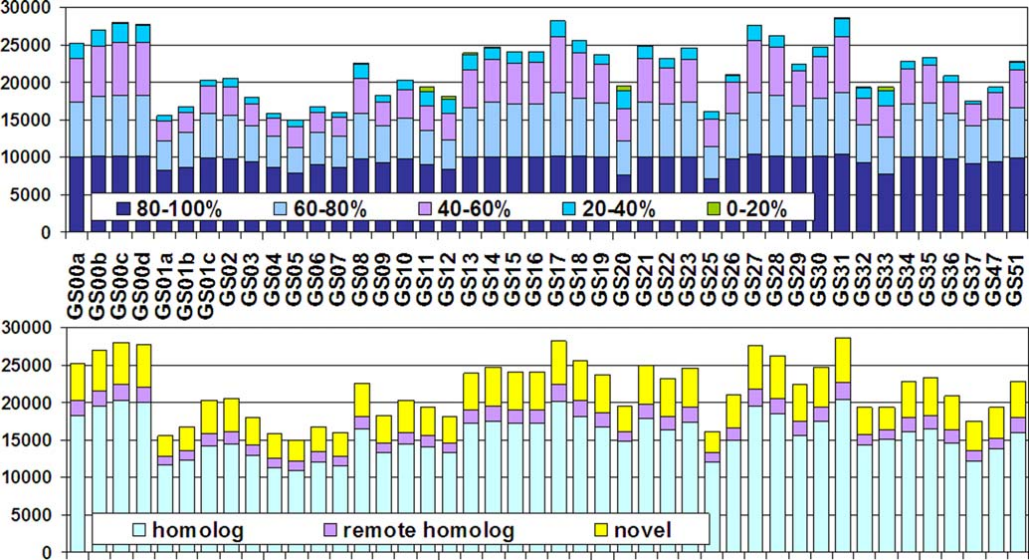
Distribution of predicted GOS protein clusters within each sample. The *y*-axis is the number of clusters. In the upper figure, clusters are grouped and colored by the percentage of samples to which a cluster is associated. In the bottom figure, clusters are colored by novelty in terms of having homologs, remote homologs, or no homolog in known protein database.

On the other hand, clusters mapped to only a small number of samples are rare; only 771 clusters are found in ≤20% samples. The top five samples containing most of these rare clusters are GS20, GS11, GS12, GS33, and GS13—each contains 628, 603, 593, 366, and 294 rare clusters. This result is not surprising because GS20 is the only freshwater sample, GS11 and GS12 are two of the three estuary samples, and GS33 is the only hypersaline sample. But it is not very obvious why this should occur for GS13, one of the 20 coastal samples. There are six completely sample-specific clusters; all of them are from a single location, the hypersaline GS033. Three of them are novel clusters; the others only have hypothetical protein homologs in the NCBI NR. In terms of the distribution of novelty of clusters, all the samples are quite similar (Figure 7) known clusters and novel clusters are distributed evenly among samples.

### Small clusters

Small clusters were not investigated in detail, mostly because of their sheer number, but also because of they are more likely spurious ORF clusters. We did repeat some of the analyses described in this paper on 49,747 clusters of sizes 10–19, which contain 971,171 ORFs. We first applied the sequence consensus measure and selected 24,119 protein-like clusters. Since there are only a small number of homologs in each cluster, this measure was not as good as when it was applied to large clusters. Therefore, we built a sequence profile for each of the 33,370 large clusters with PSI-BLAST by searching the non-redundant sequences in that cluster using the representative as the query. These profiles were used to search the ORFs in small clusters to identify matches of expect value of ≤1e-3 over 60 residues. If 50% of the ORFs in a small cluster match a big cluster profile, this small cluster is also marked as protein-like. This gave 20,718 protein-like clusters. By combining these two groups, we obtained 29,882 predicted protein clusters from these small clusters. The small clusters contain 611,046 ORFs, making a total of 6.6 million ORFs in all predicted protein clusters.

### Annotation server

We annotated the representatives of 33,370 predicted protein clusters with a list of programs including TMHMM [35], SignalP [36], Jnet [37], Coils [38], Hmmer [32], BLAST [30], PDB-BLAST [31], FFAS [33], Clustalw [39] and Modeller [40]. These programs cover trans-membrane topology prediction, signal peptide prediction, secondary structure prediction, coiled-coil prediction, low-complexity region calculation, domain identification, homology search, multiple alignments, 3D structure prediction, and so on. The results are available at http://tools.camera.calit2.net/gos/.

## Discussion

Using ultra-fast clustering followed by automated analyses of the clusters, we were able to quickly analyze a very large metagenomic dataset. Since our clustering and annotation pipeline is two orders of magnitude faster than conventional approaches, it can be easily applied to other large datasets in the future. With modifications, a similar clustering method has been used in analyzing several 454 based metagenomic datasets, which will be reported in separate studies.

Challenges still remain after this initial level of analyses. These include the further annotation and validation of novel clusters, the analysis of genes from small clusters, the identification of novel domains, and the prediction and discovery of new functions. Such tasks can only be carried out by the whole community of researchers, so we have made the results of our analysis fully available on the CAMERA Web site. Metagenomics will require extensions and optimization of existing tools and the creation of new, metagenomics-specific ones, as well as the means to do large-scale data integration of the results from separate observations and to provide informative annotation on a large scale. New approaches are needed for assembly and for binning the sequences, and a new conceptual approach to annotation, in which the community is involved through a wiki environment, will be essential.

## Methods

### Sequence clustering

The clustering algorithm behind CD-HIT is a greedy incremental algorithm. In a single clustering run, sequences are first sorted in order of decreasing length. The longest sequence becomes the representative of the first cluster. Then, each remaining sequence is compared to the representatives of all existing clusters. If the predefined similarity threshold is met, the sequence is grouped into the most similar cluster. Otherwise, a new cluster is defined with that sequence as the representative. When finished, each cluster has only one representative, the longest sequence. In this study, we performed the CD-HIT clustering three times in succession with decreasing similarity thresholds. First, clustering started with the input dataset, and the last two steps started with representatives of the previous clustering runs. The clustering of the first two steps required full-length sequence similarity, but the last step needed only >¾ coverage in length. The whole process iteratively joins the similar sequences into families and therefore produces a hierarchical structure for the input dataset. In this study, we used the newly added accurate mode for clustering. The usage can be found from the CD-HIT user's guide.

### Sequence similarity comparison

Three methods, BLASTP [30], PDB-BLAST [31], and FFAS [33], were used to compare the GOS ORFs to known protein databases. PDB-BLAST is a two-step PSI-BLAST searching method. The first run with three iterations is performed against a comprehensive database (db1) to build a Position Specific Scoring Matrix (PSSM). The second run, done without iteration, searches against the object database (db2) with the pre-calculated PSSM. In this study, db1 contains non-redundant sequences at 90% identity from GOS ORFs and NCBI NR with sequences of low-complexity composition masked. The pre-processing of db1 significantly reduces the search time and improves sensitivity. FFAS is a profile-profile comparison program. In order to compare two sequences, sequence profiles are built for both of them. The sequence profiles for queries were built based on the first step of the PDB-BLAST runs. The target profiles were provided by the FFAS developers.

### Detection of non-protein-coding ORFs with sequence consensus

A multiple alignment is built for each cluster using the representative and up to 30 of its nearest sequences with ClustalW [39]. We identified the consensus positions where the residues are conserved to ≥80% of the sequences. Residues are considered conserved if they are identical or belong to the same groups (K/R, E/D, T/S, and L/V/I) and their corresponding DNA codons are not conserved. The latter requirement ensures that the conservation is maintained by the evolutional pressure at the protein level. Let *n* be the number of codons for an amino acid *X*, the probability of each codon to be observed is 1/*n*. We do not consider *X* to be conserved if any single codon is too dominant at a level that all other *n–1* codons together are observed at less than half of the sum of their probabilities: the occurrence of other *n–1* codons<*(n–1)/2n*.

The minimal number of consensus positions required by real protein families was obtained using the Pfam seed alignments with at least 20 sequences. We found that 99% of these alignments meet follow criteria: (1) the number of consensus positions is ≥15 or ≥15% of the alignment length, and (2) consensus residues must contain ≥2 active residues (E, D, R, K, H, S, T, C, N, and Q). These criteria were used to filter out spurious ORF clusters.
